# What are the important factors influencing the physical activity level of junior high school students: a cross-sectional survey

**DOI:** 10.3389/fpubh.2024.1380290

**Published:** 2024-05-16

**Authors:** Huijun Ma, Xuefeng Li, Chengliang Ma, Da Teng

**Affiliations:** ^1^College of Physical Education, Taiyuan University of Technology, Taiyuan, China; ^2^Graduate School of Sport Science, Waseda University, Saitama, Japan; ^3^School of Physical Education, Shanxi Agriculture University, Jinzhong, China; ^4^School of Sports Medicine and Rehabilitation, Beijing Sport University, Beijing, China

**Keywords:** physical activity level, energy consumption, influencing factors, junior high school students, social ecology

## Abstract

**Background:**

Engaging in regular physical activity has been consistently linked to improved physical health and academic performance. Despite its known benefits, there is a concerning trend of decreased physical activity among children globally. The study primarily aims to investigate the level of physical activity among junior high school students in Taiyuan and analyse the main affecting factors from a socio-ecological perspective.

**Methods:**

A cross-sectional study was conducted, involving 650 junior high school students from 7 schools in Taiyuan, and 648 valid questionnaires were ultimately collected. The data on students’ physical activity levels were collected through the Children’s Leisure Activities Study Survey Questionnaire, and the data on factors affecting students’ physical activity were collected through the Student Perceived Factors Affecting Physical Activity Questionnaire.

**Results:**

In this study, students from the 7th, 8th, and 9th grades participated in physical activities, averaging 214.500 min per week in moderate-intensity and 25.000 min in high-intensity activities, cumulatively averaging 280.000 min weekly. Notably, a significant disparity (*p* = 0.012) was observed in the combined duration of moderate and high-intensity activities, with male students engaging more time compared to their female counterparts (307.500 vs. 255.000 min). This variance extended across different grades, particularly evident in 8th graders who recorded the highest weekly high-intensity activity duration (31.000 min) and overall physical activity time (320.500 min), surpassing the 7th graders(*p* = 0.007 for high-intensity activities). Furthermore, an exploratory factor analysis of a 32-item questionnaire, designed to identify determinants of physical activity, revealed six principal components. These components were found to positively correlate with both moderate and high-intensity physical activities.

**Conclusion:**

Results emphasize that educational institutions and community programs should collaborate to offer engaging weekend physical activity programs. Schools should develop and implement tailored physical education curricula addressing gender and grade differences. Furthermore, schools and local governments should invest in high-quality sports equipment and facilities.

## 1 Introduction

Physical fitness and health are paramount for the growth and development of adolescents (1). This importance is underscored by a growing body of research that links active lifestyles during adolescence with reduced risk of chronic diseases such as type 2 diabetes and cardiovascular disorders later in life (2). Regular physical activity during these formative years not only promotes physical well-being but also impacts mental health, academic performance, and social development (3). Adequate physical activity in this crucial phase lays the groundwork for lifelong health and well-being (4). Despite the clear benefits, the decline in PA among adolescents over the past decade presents a complex challenge influenced by a blend of social, environmental, and psychological factors (5). However, recent trends have sparked concerns regarding the physical health of adolescents, emerging as a pressing global issue (6). Rapid urbanization and technological advancements have contributed to a more sedentary lifestyle among this young population. According to surveys conducted in Europe and the United States, only about 15% (7) and 18.4% (8) of adolescents meet recommended levels of PA, respectively. Moreover, Herman’s research indicates that a mere 16% of adolescents maintain active physical behavior into adulthood (9). These alarming statistics highlight an urgent need for targeted interventions that can effectively address the barriers to PA specific to this age group. Unlike younger children or adults, junior high school students are in a critical transition phase, experiencing rapid physical growth, hormonal changes, and cognitive development. This period is pivotal for instilling lifelong health and fitness habits. As a result, the question of how to effectively address the declining trend in physical fitness among junior high school students remains a central issue drawing substantial attention in the realms of modern education and sports.

Previous research has indicated that the diminishing physical health of adolescents is the result of a prolonged impact from various factors (10). Among these factors, the lack of PA is widely recognized domestically and internationally as the primary cause of this decline (11). PA refers to any bodily movement produced by skeletal muscles that requires energy expenditure (12). Studies have shown that regular participation in moderate- and high-intensity physical activities offers numerous health benefits for children and adolescents, including weight management, prevention of obesity, enhanced lipid profiles, metabolic syndrome mitigation, reduced blood pressure, and improved mental well-being (13). Moreover, adopting healthy PA patterns during adolescence not only promotes growth and development but also has lasting effects into adulthood, significantly contributing to the prevention of chronic diseases and the deceleration of aging processes (14). Patnode CD and Lytle LA’s research reveals that PA is influenced by several critical factors (15). Socioeconomic status, for instance, plays a significant role, as it affects access to recreational facilities and equipment. Environmental factors, such as the availability of safe and accessible spaces for exercise, also greatly influence PA levels (15). Furthermore, psychological elements like motivation and personal attitudes towards health and fitness can drive or deter adolescent participation in physical activities (16). Family and peer influences are also crucial, as support from these groups can encourage regular engagement in exercise (17). Additionally, educational institutions play a pivotal role by integrating physical education into their curriculum and promoting an active lifestyle among students (18). Furthermore, to improve the physical health of adolescents, many countries have established guidelines for youth PA, with many recommending that children and adolescents engage in at least 60 min of moderate- to high-intensity PA on a daily basis (19, 20). These guidelines reflect a growing consensus on the need for comprehensive strategies that extend beyond school-based programs to include community and policy initiatives that support physical activity among youth.

Despite considerable advancements in PA research at both domestic and international levels, the predominant body of literature primarily resides in the domains of medicine and sports science. This literature mainly employs methods such as heart rate monitoring (21) and pedometers (22) to assess students’ PA levels, examining their relationships with various chronic diseases (23) and mental health (23). However, there remains a notable gap in comprehensive research that considers the dimensions of time, intensity, types of physical activities, and sedentary behavior. Moreover, studies focusing on intervention strategies for PA have predominantly been confined to empirical analyses at the individual level (24). In light of these observations, this study aims to fill these gaps by investigating the PA levels of junior high school students from a socio-ecological perspective. Furthermore, the research analyses key influencing factors to provide evidence for the enhancement of students’ PA levels, reduction of obesity rates, and the overall strengthening of their physical health. By considering a range of variables, this study aims to identify the significant factors associated with moderate and vigorous physical activity levels among junior high school students, enhancing our understanding of how various environments and backgrounds influence these activity intensities.

## 2 Methods

### 2.1 Study design and setting

To facilitate the survey and ensure the careful monitoring of participants, a cross-sectional study was conducted in Taiyuan City. This involved employing a random stratified cluster sampling method to select junior high school students from six districts: Yingze, Xinghualing, Xiaodian, Jiancaoping, Wanbailin, and others. The sampling frame encompassed seven schools systematically chosen from northern, central, and southern districts to ensure the study’s representativeness and fairness. The selection of sample schools and the recruitment of participants occurred between 25 and 26 December 2023, during which informed consent forms were obtained from the participants and their parents.

The study involved a total of 650 students from the first, second, and third grades of junior high school, with the objective of providing a representation of the overall PA levels within this demographic in Taiyuan City. Data collection was conducted in the student activity centres of the selected schools from 25 December 2023 to 2 January 2024. Throughout the data collection phase, two students were unable to continue participating (1 absence and 1 case of incomplete information), resulting in a final sample of 648 participants. The sample comprised 338 males (52.16%) and 310 females (47.84%), with a grade-level distribution of 34.72% in the 7th grade, 30.86% in the 8th grade, and 34.41% in the 9th grade.

Questionnaire administration was supervised by three class teachers and three research team members in each school. Their responsibilities included ensuring order during the process and providing instructions for completing the questionnaire within a designated 60 min period. Following completion, the questionnaires were collected and handed over to a designated researcher for systematic data analysis. The distribution of students from different schools is detailed in Table 1, highlighting the emphasis on achieving a balanced representation across the study’s different regions.

**Table 1:** School sample.

Title	Category	Frequency	Percent (%)	Accumulated percentage (%)
Gender	Male	338	52.16	52.16
	Female	310	47.84	100.00
Grade	7th Grade	225	34.72	34.72
	8th Grade	200	30.86	65.59
	9th Grade	223	34.41	100.00
School	1.0	99	15.28	15.28
	2.0	90	13.89	29.17
	3.0	91	14.04	43.21
	4.0	88	13.58	56.79
	5.0	69	10.65	67.44
	6.0	150	23.15	90.59
	7.0	61	9.41	100.00
Total		648	100.0	100.0

### 2.2 Participants

The inclusion criteria to participate in the study were as follows: (a) being students in Grades 7, 8, and 9 from junior high schools in Taiyuan, China and (b) not having any disease that would prevent the engagement in PA.

Prior to commencing the study, the schools, as well as the fathers, mothers, and/or guardians, were informed about the objectives of the study. Written informed consent was obtained from all participants or their legal representatives to ensure their voluntary participation. This research received approval from the Ethics Committee of the local institution under No. TYUT2023122202. The basic information regarding the participating students is outlined in Table 2.

**Table 2:** Basic information of participants.

Title	*N*	Mean	S.D.
Age (year)	648	14.347	1.012
Height (cm)	648	164.512	8.449
Weight (kg)	648	53.841	29.552

### 2.3 Variables and measures

#### 2.3.1 Physical activity levels

In this study, the PA levels of students were represented by two indicators: PA time and energy expenditure during physical activities. A questionnaire was utilized to collect the above data. Among various questionnaires, considering China’s national conditions and the aims of this study, the Children’s Leisure Activities Study Survey (CLASS) questionnaire (modified CLASS-C Questionnaire), originally from an Australian university and later revised by scholar Li Haiyan (25), was selected. This is known as the Adolescent Daily Physical Activity Survey. Upon review, it was observed that the revised questionnaire maintained the format of the original version while incorporating changes, Common activities like football, badminton, and walking were retained. Activities that are not commonly participated in by mainland Chinese students, such as rugby, golf, squash, karate, bowling, baseball, and judo, were removed. Instead, they were replaced with activities like jumping, throwing, shuttlecock kicking, broadcast gymnastics, and mat exercises, which are common in physical education classes in Chinese primary and secondary schools. Additionally, sedentary lifestyle activities, such as listening to music, taking phone calls, and artistic creation, were combined. As a result, the final questionnaire encompasses 24 types of moderate- to high-intensity physical activities and 8 types of sedentary behaviors. The questionnaire has been validated and has achieved high reliability and validity, aligning with the cognitive level of Chinese adolescents (26).

In this study, Monday to Friday is explicitly defined as weekdays, while Saturday and Sunday are defined as rest days. Additionally, the measurement of PA levels involves a critically important concept – energy expenditure. Energy expenditure is associated with PA: the more PA one engages in, the greater the energy expenditure, showing a positive correlation. The energy expenditure from PA, represented by the Metabolic Equivalent of Task (MET), is not only closely related to body weight, but as a significant component of total energy expenditure in the human body, it plays a vital role in maintaining energy balance (27). Therefore, this study employed energy expenditure from PA as one of the indicators to reflect the level of PA. The formula used to calculate the energy expenditure of PA is as Eq. 1:


(1) $$E=\frac{\left(\mathrm{Met}-1\right)\times 3.5\times W\times T}{200}$$

E: Energy Expenditure of Physical Activity (kcal); Met: Metabolic Equivalent ml/(kg·min); W:Weight (kg); T:Time (min).

The MET values used in this study primarily derive from Ainsworth et al.’s “Compendium of Physical Activities” (28). Furthermore, after consulting PA experts and reviewing standards set by the American College of Sports Medicine, the intensity of PA was categorized into moderate intensity (4.5 METs) and high intensity (7.5 METs).

#### 2.3.2 Influencing factors of physical activity

In this study, after reviewing the literature and consulting experts, the “Student Perceived Factors Affecting Physical Activity Questionnaire” developed by the Jiangsu Province Student Physical Health Promotion Research Center was chosen as the survey tool (29). The questionnaire is designed based on the content of the Social-Ecological Systems Theory. It references previous research on the factors affecting physical activity, investigating three aspects: the individual level, the interpersonal relationship level, and the school community organization level. The questionnaire employs a 5-point Likert scale for responses and scoring, with “Strongly Disagree”, “Disagree”, “Uncertain”, “Agree”, and “Strongly Agree” being scored as 1, 2, 3, 4, and 5, respectively. It captures the subjective experiences of students to understand their different states and levels of experience regarding each aspect. This questionnaire has been tested and achieved high reliability and validity (30).

### 2.4 Study size

The total in-school population of junior high students in Taiyuan was approximately 100,000 (31). Based on the “Table for Determining Sample Size from a Given Population” (32), the required sample size for the survey was calculated to be 384 students. However, to ensure a representative sample across all six districts of Taiyuan, at least one school was selected from each district, leading to a final sample size of 650 students from three grades. This number significantly exceeds the minimum required sample size of 384, thereby enhancing the study’s representativeness and the reliability of its findings.

### 2.5 Statistical methodology

SPSS 23.0 software was used for statistical analysis. For non-normally distributed data, the median M (P25, P75) was used for representation. The Mann–Whitney test was applied to analyse the differences in PA time and energy expenditure between genders. The Kruskal–Wallis test was utilized to examine variations in PA time and energy expenditure across different grades. An exploratory factor analysis was conducted to assess the dimensions and validity of factors perceived by students to influence PA, employing principal component analysis for extraction and varimax rotation for factor rotation. Pearson correlation analysis was used to examine the relationship between various factors and PA time and energy expenditure. A *p*-value of less than 0.05 was considered statistically significant.

## 3 Results

### 3.1 Physical activity level of students

#### 3.1.1 Physical activity time and energy expenditure

The results about PA time and energy expenditure of students are presented in Table 3. The average time spent in moderate-intensity PA for students was 129.000 min, high-intensity PA was 10.000 min, and the combined moderate- and high-intensity activity time averaged 172.500 min on weekdays. On weekends, the time spent in moderate-intensity PA averaged 70.000 min, high-intensity PA averaged 0 min, and the combined time averaged 90 min, which was lower compared to weekdays where the combined moderate- and high-intensity activity time averaged 172.500 min. Over a week, the time spent in moderate-intensity PA averaged 214.500 min, high-intensity PA averaged 25.000 min, and the combined time averaged 280.000 min, with weekly time spent on moderate-intensity physical activity being higher than that spent on high-intensity activity. The average weekly energy expenditure for moderate-intensity PA was 681 kcal, and for HIPA, it was 147 kcal.

**Table 3:** Physical activity time and energy expenditure.

Time	Title	*N*	Min.	Max.	Mean	S.D.	Median
Weekday	Moderate-intensity physical activity time (min)	648	0.000	980.000	161.204	142.446	129.000
	High-intensity PA time	648	0.000	980.000	53.199	97.012	10.000
	Moderate- and high-intensity PA time (min)	648	0.000	1410.000	214.403	180.434	172.500
Weekend	Moderate-intensity PA time (min)	648	0.000	2150.000	94.227	123.989	70.000
	High-intensity PA time	648	0.000	1080.000	29.472	74.793	0.000
	Moderate- and high-intensity PA time (min)	648	0.000	2150.000	123.699	149.714	90.000
A week	Moderate-intensity PA time (min)	648	0.000	2950.000	255.431	222.652	214.500
	High-intensity PA time	648	0.000	1735.000	82.671	154.313	25.000
	Moderate- and high-intensity PA time (min)	648	0.000	2950.000	338.102	279.533	280.000
A week	Moderate-intensity energy consumption	648	0.000	15055.000	852.319	942.496	681.000
	High-intensity energy consumption (kcal)	648	0.000	30276.000	554.904	1520.121	147.000

#### 3.1.2 Comparison of physical activity time and energy expenditure based on gender

**Figure 1:**
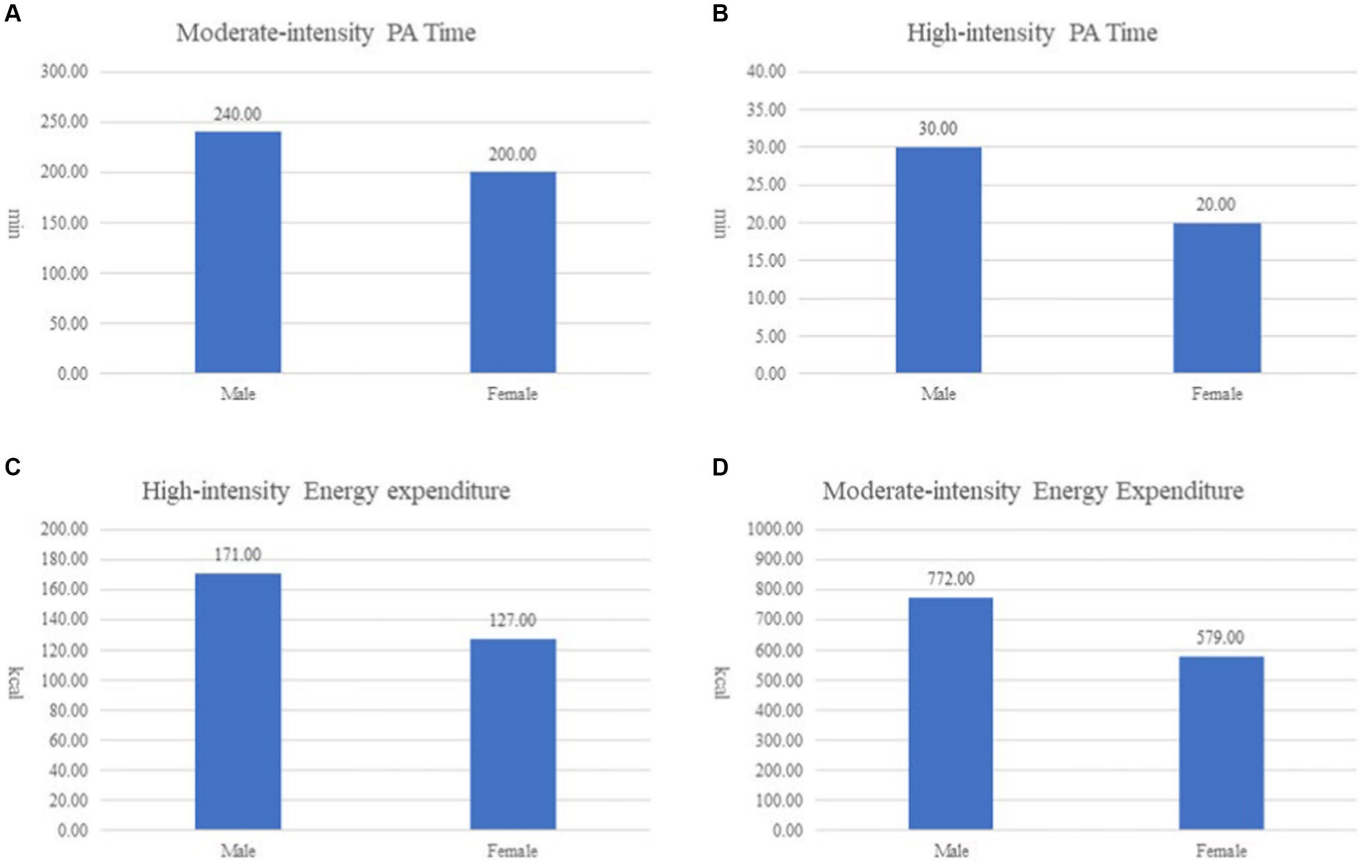
**(A)** Gender differences in moderate-intensity PA time; **(B)** Gender differences in high-intensity PA time; **(C)** Gender differences in high-intensity energy expenditure; **(D)** Gender differences in moderate-intensity energy expenditure.

The results about gender differences in PA time and energy expenditure are presented in Table 4. Non-parametric tests were employed to investigate the differences in weekly moderate-intensity, high-intensity, and combined moderate- and high-intensity PA time among students of different genders. The analysis with the Mann–Whitney U test indicated no significant differences (*p* > 0.05) in moderate-intensity time (Figure 1A) and high-intensity activity time (Figure 1B), showing consistency across genders. However, a significant difference (*p* < 0.05) was observed in combined moderate- and high-intensity activity, with male students having a higher median time (307.500) than female students (255.000).

**Table 4:** Comparison of weekly PA time and weekly PA energy expenditure by gender.

	Gender median M (P_25_, P_75_)		U	*z*	*p*-value
	Male (*n* = 338)	Female (*n* = 310)			
Moderate-intensity PA time (min)	240.000 (117.5, 350.0)	200.000 (98.8, 341.3)	48732.500	−1.537	0.124
High-intensity PA time (min)	30.000 (0.0, 130.0)	20.000 (0.0, 90.0)	49260.500	−1.373	0.170
Moderate- and high-intensity PA time (min)	307.500 (170.0, 466.3)	255.000 (131.5, 412.0)	46414.500	−2.510	0.012*
Moderate-intensity energy Expenditure (kcal)	772.000 (362.3, 1203.3)	579.000 (292.5, 1019.3)	45048.500	−3.084	0.002**
High-intensity energy expenditure (kcal)	171.000 (0.0, 812.3)	127.500 (0.0, 512.0)	48467.000	−1.720	0.085

* *p* < 0.05, ** *p* < 0.01.

Non-parametric tests were conducted to examine the differences in weekly moderate-intensity and high-intensity energy expenditure between male and female students. The analysis using the Mann–Whitney U test revealed no significant difference (*p* > 0.05) in high-intensity energy expenditure (Figure 1C), indicating consistency across genders. However, there was a significant difference (*p* < 0.05) in moderate-intensity energy expenditure (Figure 1D), with male students showing a higher median value (772.000) compared to female students (579.000), indicating gender differences in this aspect.

#### 3.1.3 Comparison of physical activity time and energy expenditure based on grades

From Table 5, which illustrates the different time spent in weekly PA by grade, using non-parametric tests, we observed the differences in weekly moderate-intensity, high-intensity, and combined moderate- and high-intensity PA times among students of different grades. The Kruskal–Wallis test showed no significant difference (*p* > 0.05) in moderate-intensity activity across grades, indicating consistency. However, significant differences were observed in high-intensity and combined moderate- and high-intensity activities (*p* < 0.05), suggesting variability across grades. Specifically, 8th grade students showed the highest weekly high-intensity PA time (31.000) and combined moderate- with high-intensity PA time (320.500), compared to 7th grade students who had the least in these categories.

**Table 5:** Comparison of weekly PA time and weekly energy expenditure by grade.

	Grade median M (P_25_, P_75_)			H	*p*-value
	7th grade (*n* = 225)	8th grade (*n* = 200)	9th grade (*n* = 223)		
Moderate-intensity PA time	195.000 (100.0, 329.5)	248.000 (100.0, 378.8)	220.000 (110.0, 350.0)	3.446	0.179
High-intensity PA time	0.000 (0.0, 80.0)	31.000 (0.0, 110.0)	30.000 (0.0, 140.0)	9.791	0.007**
Moderate- and high-intensity PA time	240.000 (132.5, 405.0)	320.500 (157.0, 490.0)	295.000 (165.0, 465.0)	9.203	0.010*
Moderate-intensity energy consumption	588.000 (291.0, 945.5)	774.000 (313.8,1212.3)	735.000 (364.0, 1211.0)	8.350	0.015*
High-intensity energy consumption	0.000 (0.0, 405.0)	186.000 (0.0, 651.8)	177.000 (0.0, 802.0)	10.915	0.004**

* *p* < 0.05, ** *p* < 0.01.

The non-parametric tests revealed significant differences in weekly moderate- and high-intensity energy consumption among students of different grades. The Kruskal–Wallis test showed significant variation (*p* < 0.05) across grades for both types of energy consumption. Specifically, 8th-grade students had the highest median weekly energy expenditure for moderate-intensity (774.000) and high-intensity (186.000) activities, whereas 7th-grade students had the lowest in these categories (588.000 and 0.000, respectively).

**Figure 2:**
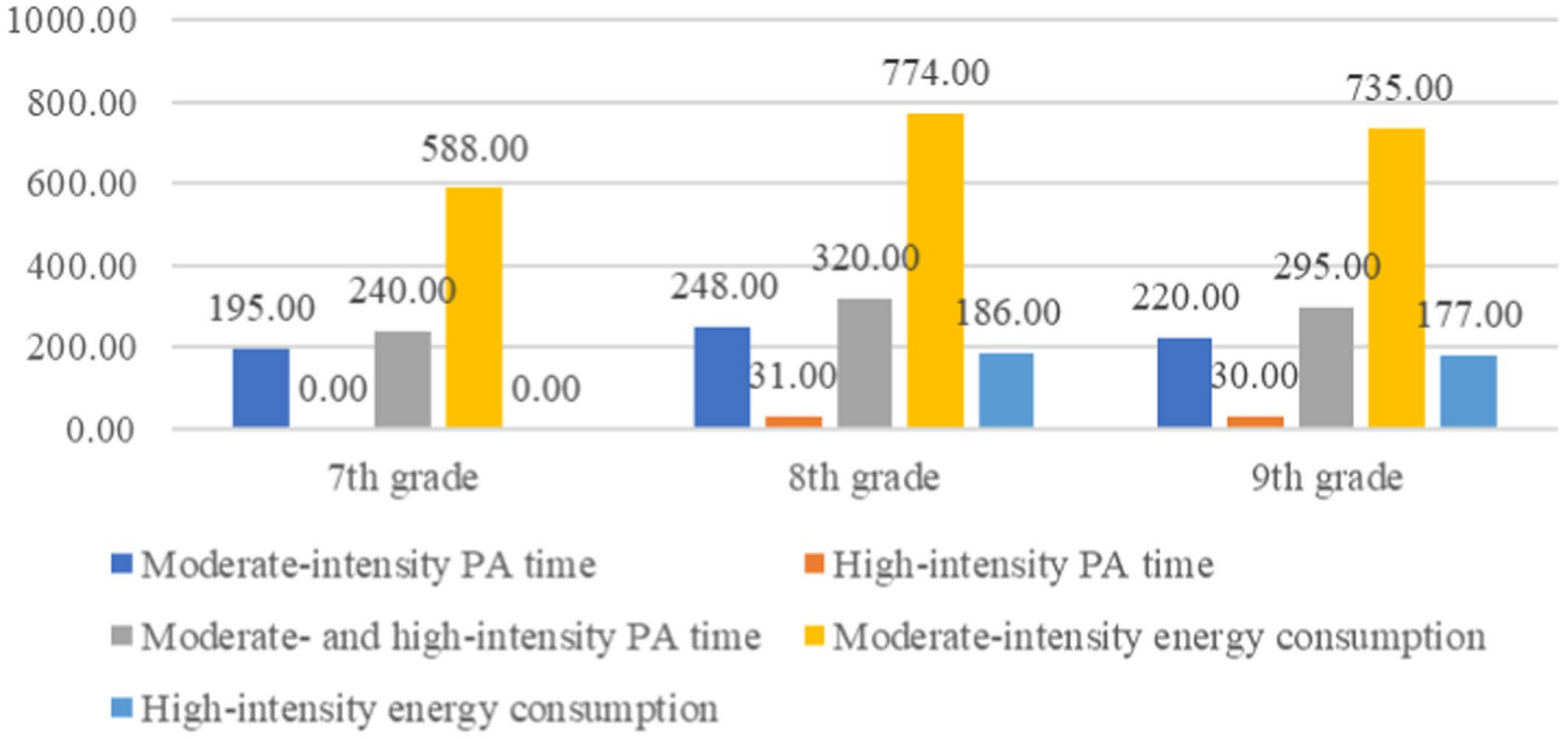
Comparison of weekly PA time and weekly energy expenditure by grade.

The non-parametric tests revealed significant differences in weekly moderate- and high-intensity energy consumption among students of different grades. The Kruskal–Wallis test showed significant variation (*p* < 0.05) across grades for both types of energy consumption. Specifically, 8th-grade students had the highest median weekly energy expenditure for moderate-intensity (774.000) and high-intensity (186.000) activities, whereas 7th-grade students had the lowest in these categories (588.000 and 0.000, respectively). We can also see form Figure 2 which clearly showed the comparison of weekly PA time and weekly energy expenditure based on grades.

### 3.2 Factors influencing students’ physical activity levels

The exploratory factor analysis was conducted on the questionnaire assessing the factors influencing students’ PA. The results are as follows: Kaiser-Meyer-Olkin = 0.908, indicating good data adequacy for factor analysis. Bartlett’s Test of Sphericity resulted in an approximate chi-square of 20718.591 (df = 496, *p* < 0.000), confirming the suitability of the data for factor analysis.

The exploratory factor analysis of the 32-item questionnaire assessing factors influencing PA resulted in the extraction of six principal components, as shown in Table 6. These components, after rotation, accounted for a total cumulative variance of 78.289%. Each factor with an eigenvalue greater than 1 was retained, indicating that these six factors were significant in explaining the variability in the data. The extraction of these six factors demonstrated an effective and substantial interpretation of the original data, with a high cumulative percentage of variance explained.

**Table 6:** Total variance explained.

Factor	Eigen			% of variance (unrotated)			% of variance (rotated)		
	Eigen value	% of variance	Cumulative % of variance	Eigen value	% of variance	Cumulative % of variance	Eigen value	% of variance	Cumulative % of variance
1	9.307	29.084	29.084	9.307	29.084	29.084	7.408	23.151	23.151
2	5.539	17.310	46.393	5.539	17.310	46.393	5.204	16.263	39.414
3	3.431	10.720	57.114	3.431	10.720	57.114	3.919	12.245	51.659
4	2.744	8.575	65.689	2.744	8.575	65.689	3.238	10.118	61.777
5	2.347	7.334	73.023	2.347	7.334	73.023	2.645	8.265	70.042
6	1.685	5.266	78.289	1.685	5.266	78.289	2.639	8.247	78.289
7	0.626	1.955	80.244	–	–	–	–	–	–
8	0.581	1.816	82.060	–	–	–	–	–	–
9	0.546	1.706	83.767	–	–	–	–	–	–
10	0.463	1.448	85.215	–	–	–	–	–	–
11	0.444	1.387	86.601	–	–	–	–	–	–
12	0.424	1.325	87.926	–	–	–	–	–	–
13	0.413	1.290	89.216	–	–	–	–	–	–
14	0.378	1.181	90.397	–	–	–	–	–	–
15	0.317	0.992	91.389	–	–	–	–	–	–
16	0.312	0.974	92.362	–	–	–	–	–	–
17	0.293	0.917	93.279	–	–	–	–	–	–
18	0.258	0.807	94.087	–	–	–	–	–	–
19	0.233	0.727	94.813	–	–	–	–	–	–
20	0.214	0.669	95.483	–	–	–	–	–	–
21	0.199	0.621	96.104	–	–	–	–	–	–
22	0.185	0.578	96.682	–	–	–	–	–	–
23	0.180	0.564	97.246	–	–	–	–	–	–
24	0.153	0.479	97.725	–	–	–	–	–	–
25	0.124	0.388	98.112	–	–	–	–	–	–
26	0.120	0.376	98.488	–	–	–	–	–	–
27	0.113	0.354	98.843	–	–	–	–	–	–
28	0.094	0.295	99.138	–	–	–	–	–	–
29	0.077	0.240	99.378	–	–	–	–	–	–
30	0.073	0.228	99.605	–	–	–	–	–	–
31	0.070	0.218	99.823	–	–	–	–	–	–
32	0.057	0.177	100.000	–	–	–	–	–	–

The factor loading coefficients represented the correlation between factors and analytical items. As shown in Table 7, the rotated factor loadings were significantly greater than 0.4, and most were above 0.7, indicating strong correlations. The commonality of each item, or the common factor variance, was also greater than 0.4, signifying good validity. The distribution of the items across the factors was as follows:

- Items 1–4 correspond to Factor 5, comprising 4 items, and were named “Equipment Support”.
- Items 5–10 correspond to Factor 3, comprising 6 items, and were named “School Organization”.
- Items 11–16 correspond to Factor 2, comprising 6 items, and were named “Parental and Peer Support”.
- Items 17–19 correspond to Factor 6, comprising 3 items, and were named “Class Teacher Factors”.
- Items 20–23 correspond to Factor 4, comprising 4 items, and were named “Physical Education Teacher Factors”.
- Items 24–32 correspond to Factor 1, comprising 9 items, and were named “Individual Cognition”.

**Table 7:** Factor loading (rotated).

Name	Factor loading						Commonality (common factor variance)
	Factor1	Factor2	Factor3	Factor4	Factor5	Factor6	
A1	0.037	0.001	0.060	0.076	**0.791**	0.089	0.645
A2	0.020	0.087	0.021	0.057	**0.852**	0.062	0.741
A3	0.045	0.165	0.055	0.126	**0.753**	−0.021	0.615
A4	0.061	0.143	0.006	−0.038	**0.787**	0.046	0.647
A5	0.011	0.025	**0.760**	0.055	0.061	0.013	0.586
A6	0.014	0.079	**0.826**	0.003	0.014	0.065	0.693
A7	0.057	0.111	**0.771**	0.064	−0.023	0.080	0.621
A8	0.056	0.073	**0.870**	0.042	0.039	0.037	0.770
A9	0.248	0.082	**0.805**	0.023	0.058	0.008	0.721
A10	0.020	0.087	**0.723**	0.045	0.020	0.033	0.534
A11	0.125	**0.908**	0.079	0.170	0.091	0.116	0.898
A12	0.114	**0.922**	0.065	0.118	0.116	0.043	0.896
A13	0.089	**0.882**	0.115	0.160	0.086	0.086	0.839
A14	0.111	**0.930**	0.081	0.117	0.068	0.051	0.904
A15	0.119	**0.900**	0.077	0.117	0.096	0.045	0.855
A16	0.133	**0.809**	0.153	0.197	0.082	0.186	0.776
A17	0.050	0.134	0.084	0.199	0.060	**0.931**	0.937
A18	0.067	0.125	0.092	0.118	0.076	**0.935**	0.923
A19	0.071	0.169	0.062	0.333	0.074	**0.835**	0.852
A20	0.030	0.146	0.055	**0.821**	0.072	0.192	0.741
A21	0.071	0.258	0.023	**0.839**	0.082	0.110	0.796
A22	0.052	0.184	0.083	**0.862**	0.073	0.107	0.804
A23	0.047	0.155	0.073	**0.873**	0.025	0.186	0.829
A24	**0.911**	0.163	0.067	0.027	0.007	0.051	0.865
A25	**0.924**	0.136	0.064	0.036	0.011	0.077	0.884
A26	**0.847**	0.178	0.010	0.064	0.034	0.040	0.756
A27	**0.928**	−0.055	0.020	−0.021	−0.004	−0.012	0.865
A28	**0.805**	−0.014	0.100	0.076	0.045	0.047	0.668
A29	**0.933**	0.080	0.034	−0.011	−0.018	0.031	0.880
A30	**0.894**	0.104	0.043	0.039	0.069	−0.009	0.819
A31	**0.899**	0.075	0.062	0.034	0.077	0.013	0.824
A32	**0.915**	0.136	0.092	0.047	0.044	0.034	0.870

Bold values in the table are all greater than 0.7.

This factor structure effectively categorized the various aspects influencing students’ perception of PA.

### 3.3 Correlation between factors and physical activity

**Figure 3:**
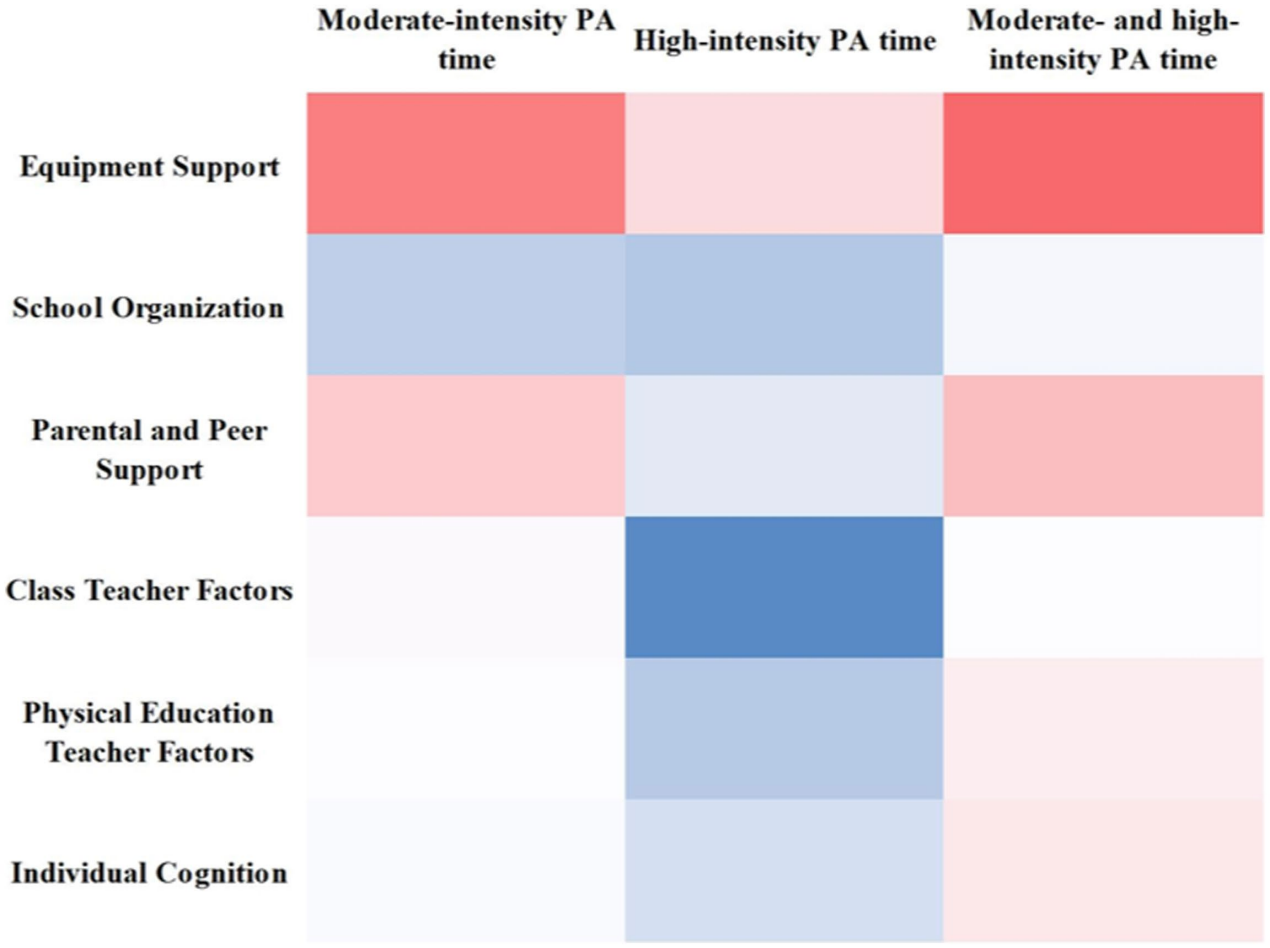
The relationships between energy expenditure at moderate and high intensities and six factors (equipment support, school organization, parental and peer support, class teacher factors, physical education teacher factors, and individual cognition).

The analysis of the relationship between moderate- and high-intensity PA time and six factors (Equipment Support, School Organization, Parental and Peer Support, Class Teacher Factors, Physical Education Teacher Factors, and Individual Cognition) using Pearson correlation coefficients revealed significant findings, as shown in Table 8. At the same time, the Figure 3 also showed the degree of correlation, which is represented by a heatmap (where the redder colors indicate higher correlation and the bluer colors indicate lower correlation):

**Table 8:** Correlation between different factors and weekly PA time.

	Moderate-intensity PA time	High-intensity PA time	Moderate- and high-intensity PA time
Equipment support	0.481**	0.268**	0.531**
School organization	0.141**	0.131**	0.185**
Parental and peer support	0.305**	0.169**	0.336**
Class teacher factors	0.199**	0.059	0.191**
Physical education teacher factors	0.191**	0.134**	0.226**
Individual cognition	0.189**	0.159**	0.238**

* *p* < 0.05, ** *p* < 0.01.

Moderate-Intensity PA Time: There was a significant positive correlation with all six factors. The correlation coefficients were 0.481 (Equipment Support), 0.141 (School Organization), 0.305 (Parental and Peer Support), 0.199 (Class Teacher Factors), 0.191 (Physical Education Teacher Factors), and 0.189 (Individual Cognition). All coefficients were greater than 0, indicating a positive relationship.

High-Intensity PA Time: This also showed a significant positive correlation with five of the factors, with coefficients of 0.268 (Equipment Support), 0.131 (School Organization), 0.169 (Parental and Peer Support), 0.134 (Physical Education Teacher Factors), and 0.159 (Individual Cognition). However, there was no significant correlation with Class Teacher Factors, as the coefficient was close to 0.

Combined Moderate- and High-Intensity PA Time: There was a significant positive correlation with all six factors. The coefficients were 0.531 (Equipment Support), 0.185 (School Organization), 0.336 (Parental and Peer Support), 0.191 (Class Teacher Factors), 0.226 (Physical Education Teacher Factors), and 0.238 (Individual Cognition), all greater than 0, indicating a positive relationship.

These results suggest that both moderate- and high-intensity physical activities were positively influenced by the mentioned factors, with varying degrees of correlation.

Table 9 presents a correlation analysis exploring the relationships between energy expenditure at moderate and high intensities and six factors (Equipment Support, School Organization, Parental and Peer Support, Class Teacher Factors, Physical Education Teacher Factors, and Individual Cognition) using Pearson correlation coefficients.

**Table 9:** Correlation between different factors and weekly energy consumption.

	Moderate-intensity energy consumption	High-intensity energy consumption
Equipment support	0.362**	0.155**
School organization	0.106**	0.071
Parental and peer support	0.261**	0.141**
Class teacher factors	0.162**	0.035
Physical education teacher factors	0.153**	0.092*
Individual cognition	0.114**	0.058

* *p* < 0.05, ** *p* < 0.01.

Significant positive correlations were found between moderate-intensity energy expenditure and various factors, including Equipment Support (0.362), School Organization (0.106), Parental and Peer Support (0.261), Class Teacher Factors (0.162), Physical Education Teacher Factors (0.153), and Individual Cognition (0.114), all significant at the 0.01 level.

In high-intensity energy expenditure, significant positive correlations were observed with Equipment Support (0.155) and Parental and Peer Support (0.141), both significant at the 0.01 level, and with Physical Education Teacher Factors (0.092), significant at the 0.05 level. No significant correlations were found with School Organization, Class Teacher Factors, and Individual Cognition.

## 4 Discussion

This study revealed the PA levels and influencing factors of 648 junior high school students in Taiyuan City. The findings highlight significant variations in the PA times and energy expenditures of students between weekdays and weekends. Notably, students engaged in more moderate- and high-intensity physical activities on weekdays compared to weekends. This observation may be partially explained by the structured nature of school environments, where scheduled physical education classes and recess promote regular activity during weekdays (33). This pattern aligns with prior research indicating reduced PA among children during non-school days (34). However, the level of high-intensity PA, especially during weekends, is lower throughout the week, which differs from some research results in similar population environments. It is also possible that the availability of parental supervision or participation during weekends varies, affecting the intensity and type of activities students can engage in (35). These differences may be attributed to regional differences in lifestyle, school curriculum, or accessibility to recreational facilities (36). In addition, the daily PA time of students does not meet the World Health Organization’s recommended guidelines for physical activities for this age group to engage in at least 60 min of moderate to vigorous PA each day (37), emphasizing the need for targeted interventions to improve PA levels, especially on weekends. Moreover, the discrepancy between actual and recommended PA levels underscores the importance of integrating health education into the school curriculum, ensuring that students understand the benefits of maintaining an active lifestyle.

The comparison of PA time and energy expenditure among junior high school students revealed gender differences. While there is no significant difference in moderate- and high-intensity PA time across genders, a notable disparity exists in the combined moderate- and high-intensity activity time, with boys showing higher levels. This suggests that while the total time spent on physical activities may be similar, boys tend to participate in activities that are more intense or require greater energy output than those typically chosen by girls. Furthermore, a significant difference in moderate-intensity energy expenditure was observed, again favouring boys. These findings highlight gender disparities in PA habits among students, suggesting that boys engage in more prolonged and energetically demanding activities. This contrasts with some studies that report minimal gender differences in activity levels among children, indicating that cultural, environmental, and educational factors might play a role in these disparities (38). These findings underscore the need for gender-specific strategies to promote PA among students.

The results also showed significant grade-based differences in PA time and energy expenditure among the students. While there is no significant disparity in moderate-intensity PA time across grades, substantial differences are noted in high-intensity and combined moderate- and high-intensity activity time, with Grade 8 students showing the highest levels. Additionally, there are significant differences in both moderate- and high-intensity energy consumption among grades, with Grade 8 students also leading in energy expenditure. This can be attributed to several factors, including developmental changes, where Grade 8 students are at a peak growth stage, leading to higher energy and activity levels (39). Furthermore, social factors and peer influence could play a role, as students in this age group may be more motivated to participate in physical activities for social interaction and skill development. Additionally, the competitive environment and peer group dynamics in higher grades may incentivize students to engage more actively in physical activities, particularly as they seek to establish social hierarchies and gain peer acceptance through sports and other physical pursuits (40). The study also found that Grade 9 students have higher levels of PA than Grade 7 students, possibly due to Grade 9 students allocating more time to academic preparations, including physical education tests, which are part of the middle school examination system (41). This shift in focus reflects the impact of national education policies, highlighting the importance of policy-driven changes in student behavior and priorities.

This study utilized exploratory factor analysis to identify factors influencing students’ PA. Six principal components are extracted, representing different aspects such as “Equipment Support”, “School Organization”, and “Individual Cognition”. This structure categorizes the various elements impacting students’ perception of PA. It is notable that each factor, such as “Parental and Peer Support”, shows a strong correlation with specific questionnaire items, underlining the complexity of factors influencing PA among students. Moreover, the interaction between these factors provides insights into the dynamic interplay of environmental, social, and personal influences that shape PA patterns among students. This comprehensive categorization aids in understanding the multifaceted nature of student PA behavior.

The findings also revealed significant positive correlations between various factors and the weekly PA levels of students. Notably, Equipment Support emerges as a consistent and significant factor in influencing moderate- and high-intensity PA time, as well as energy expenditure. This corroborates previous research underscoring the critical role of accessible and quality equipment in promoting physical engagement (42). According to the self-determination theory by Deci and Ryan (43), the presence of high-quality sports equipment may enhance the perceived competence and autonomy among students, which are vital for fostering intrinsic motivation towards engaging in physical activities. In contrast, Class Teacher Factors showed a weaker, albeit still positive, correlation. This could be attributed to the variable influence of classroom teachers on PA, as suggested by Trigueros et al. (44). Interestingly, this factor did not significantly correlate with high-intensity PA, potentially indicating a threshold in the impact of classroom teaching methods on more vigorous forms of exercise. The moderate correlations with Parental and Peer Support, as well as Individual Cognition, highlight the importance of social and psychological elements in PA engagement, as discussed by Haidar et al. (45), who emphasized the role of social and cognitive factors in shaping PA patterns among youth. Furthermore, the influence of School Organization, while significant, is relatively modest. This finding is in partial agreement with previous research (46), which suggested that institutional factors might not be the primary drivers of energy expenditure in physical activities.

Overall, the findings corroborate the existing literature while providing new insights into the differential impacts of various factors on PA intensity. Specifically, the analysis highlights the unique contribution of equipment quality and availability in motivating students. This revelation points to practical applications in school policy where investments in high-quality sports equipment could be prioritized. Furthermore, the interplay between teacher roles and social supports, such as peer and parental encouragement, suggests a multi-faceted strategy is necessary to effectively enhance PA levels among junior high students. Educational programs should not only focus on the physical resources but also on cultivating an encouraging social environment.

### 4.1 Limitations

This study has several limitations that should be mentioned. Firstly, as a cross-sectional study, this research involved data collection over a brief period, making it challenging to discern causal relationships between PA and its impact on physical fitness, cardiopulmonary endurance, and bone health. The study primarily facilitated a correlation analysis between certain factors. Consequently, future research would benefit from more comprehensive longitudinal tracking studies that explore these relationships in greater depth. Secondly, compared to accelerometers, PA questionnaires provide a more rudimentary measurement and yield less precise data. Therefore, it is recommended that future studies combine accelerometers with questionnaires to enhance measurement accuracy. Thirdly, the analysis of influencing factors in this study is not thorough enough. For instance, motivation is a critical determinant of PA, although it is briefly addressed in this manuscript. Based on the self-determination theory (43), in a recent research by Ahmadi et al. (47), a classification system of motivational behaviors has been proposed. Future research could build on this classification system to further develop and test an intervention program aiming at enhancing motivation towards physical activity levels among adolescents.

## 5 Conclusion

This study investigated the PA levels and main influencing factors among junior high school students in Taiyuan, providing a theoretical basis for improving the level of PA of students and enhancing the physical fitness of students. The research indicates that students engage more in moderate- and high-intensity activities on weekdays, with a marked decline during weekends. Notably, the daily PA time of students does not meet the World Health Organization’s recommended guidelines, especially on weekends. Additionally, the study underscored gender- and grade-based disparities in PA, with boys and Grade 8 students showing higher levels of activity and energy expenditure. This emphasized the need for age- and gender-specific PA guidelines and interventions in schools. Furthermore, the study has identified six key influencing factors of PA among students, including “Equipment Support”, “School Organization”, “Parental and Peer Support”, “Class Teacher Factors”, “Physical Education Teacher Factors”, and “Individual Cognition”, which play important roles in shaping students’ PA perceptions and behaviors. In addition, this study underscored the pivotal role of Equipment Support in promoting moderate- to high-intensity physical activities and energy expenditure and highlighted the lesser, yet positive, impact of Class Teacher Factors, alongside the moderate influence of Parental and Peer Support and Individual Cognition. These insights emphasize the importance of a comprehensive approach to physical education, integrating equipment availability, educational practices, and socio-cognitive elements.

### 5.1 Implications

This study has significant implications for various educational and health-related fields. First, it underscores the urgent need for targeted policies to boost PA during weekends when a marked decline is observed. It is suggested that educational institutions and community programs collaborate to offer engaging weekend PA programs. Second, the highlighted gender- and grade-based disparities call for the development of age- and gender-specific PA guidelines in schools. This suggests that schools should develop and implement tailored physical education curricula that address these differences. Furthermore, the study’s identification of six key factors influencing PA emphasizes the necessity of multifaceted interventions. Additionally, the pivotal role of Equipment Support in promoting physical activities cannot be overlooked, necessitating enhanced infrastructure in educational institutions. It is suggested that schools and local governments invest in high-quality sports equipment and facilities. Finally, the study advocates for an integrated approach in physical education, combining equipment availability with educational and socio-cognitive elements, to holistically improve student fitness and engagement in physical activities.

## Data availability statement

The raw data supporting the conclusions of this article will be made available by the authors, without undue reservation.

## Ethics statement

The studies involving humans were approved by the Biomedical Ethics Committee of the Academic Committee of Taiyuan University of Technology. The studies were conducted in accordance with the local legislation and institutional requirements. The participants provided their written informed consent to participate in this study.

## Author contributions

HM: Conceptualization, Funding acquisition, Investigation, Methodology, Writing – original draft, Writing – review & editing. XL: Conceptualization, Data curation, Resources, Supervision, Writing – review & editing. CM: Investigation, Validation, Writing – original draft. DT: Methodology, Writing – review & editing.
