# Corrigendum: What are the important factors influencing the physical activity level of junior high school students: a cross-sectional survey

**DOI:** 10.3389/fpubh.2025.1565996

**Published:** 2025-02-06

**Authors:** Huijun Ma, Xuefeng Li, Chengliang Ma, Da Teng

**Affiliations:** ^1^College of Physical Education, Taiyuan University of Technology, Taiyuan, China; ^2^Graduate School of Sport Science, Waseda University, Saitama, Japan; ^3^School of Physical Education, Shanxi Agriculture University, Jinzhong, China; ^4^School of Sports Medicine and Rehabilitation, Beijing Sport University, Beijing, China

**Keywords:** physical activity level, energy consumption, influencing factors, junior high school students, social ecology

In the published article, there were two errors in the reporting of the data collection dates.

A correction has been made to **2 Methods**, *2.1 Study design and setting*, Paragraph 1. This sentence previously stated:

“The selection of sample schools and the recruitment of participants occurred between 10 and 14 December 2022, during which informed consent forms were obtained from the participants and their parents.”

The corrected sentence appears below:

“The selection of sample schools and the recruitment of participants occurred between 25 and 26 December 2023, during which informed consent forms were obtained from the participants and their parents.”

Another correction has been made to **2 Methods**, *2.1 Study design and setting*, Paragraph 2. This sentence previously stated:

“Data collection was conducted in the student activity centres of the selected schools from 15 to 22 December 2022.”

The corrected appears below:

“Data collection was conducted in the student activity centres of the selected schools from 25 December 2023 to 2 January 2024.”

The authors apologize for this error and state that this does not change the scientific conclusions of the article in any way. The original article has been updated.

